# Are peripherally inserted central catheters in critical ill patients justified?

**DOI:** 10.1186/2197-425X-3-S1-A72

**Published:** 2015-10-01

**Authors:** O Martínez González, D Ballesteros, C Martín Parra, M Chana, B López Matamala, MA Alonso Fernández, B Estébanez, J Luján, R Blancas

**Affiliations:** Critical Care, Hospital Universitario del Tajo, Aranjuez, Spain; Critical Care, Hospital Universitario la Paz, Madrid, Spain; Critical Care, Hospital Universitario Príncipe de Asturias, Alcalá de Henares, Spain

## Introduction

Peripherally inserted central catheters are an alternative to central venous catheters in critically ill patients. In recent years its use is increasing despite first central access choice in these patients are central venous catheters.

## Objectives

To describe if peripherally inserted central catheters are justified to be cannulated instead central venous catheters. Secondary objective is to describe the duration and time of cannulation related to admission time to the ICU.

## Methods

A retrospective study conducted in the ICU of the Hospital Universitario del Tajo from 2008 to 2014. All peripherally inserted central catheters were collected, date of cannulation and removal, and the presence at the cannulation time of antiplatelet therapy, anticoagulation therapy (INR > 1.4), coagulopathy (INR > 1.4, prothrombin T+time(PT) > 60 sec, partial thromboplastin time (PTT) > 45 sec), thrombocytopenia ( < 100000/mm^3^), anatomical alterations contraindicating central venous catheters cannulation, decubitus intolerance and/or obesity (BMI> 35). For the statistical analysis SPSS v.20.0 software was used, using percentages to describe quantitative variables and means, or medians, to describe quantitative variables.

## Results

204 peripherally inserted central catheters were cannulated. The median duration was 3 days (CI 25% -75%: 2-4). Only 108 catheters, 58.9% (95% CI:46,3%-60,1%), had justification for cannulation instead of central venous catheters. The most frequent causes were coagulopathy in 47 patients (23.0%) and antiplatelet therapy in 33 (16.2%) (Table 1). Most of the peripherally inserted central catheters cannulated were at the afternoon shift (15:00-22:00) being this setting 91 catheters, 37.9% (95% CI: 44,2%-51,2%).

**Table 1:** Table 1

	Number of patients	Percentage	Lower limit (CI 95%)	Upper Limit (CI 95%)
Obesity(BMI>35)	19	9,3	6	14,1
Antiplatelet therapy	33	16,2	11,8	21,8
Anticoagulation Therapy	30	14,7	10,5	20,2
Coagulopathy	47	23	17,8	29,3
Thrombocytopenia	6	2,9	1,3	6,3
Anatomical Alterations	1	0,5	0	2,7
Decubitus intolerance	23	11,3	7,6	16,3

## Conclusions

Cannulation peripheral inserted central catheters requires further justification now at ICU. Pending further studies, central venous catheters is now the first central access choice in critically ill patient, so peripheral inserted central catheters is an alternative in selected patients with risk factors to increase incidence of complications.

